# COVID‐19 retreats and world recovers: A silver lining in the dark cloud

**DOI:** 10.1002/hcs2.57

**Published:** 2023-08-08

**Authors:** Amol Chhatrapati Bisen, Sristi Agrawal, Sachin Nashik Sanap, Heamanth Ganesan Ravi Kumar, Nelam Kumar, Rajdeep Gupta, Rabi Sankar Bhatta

**Affiliations:** ^1^ Pharmaceutics and Pharmacokinetics Division CSIR—Central Drug Research Institute Lucknow Uttar Pradesh India; ^2^ Academy of Scientific and Innovative Research (AcSIR) Ghaziabad Uttar Pradesh India; ^3^ Biochemistry and Structural Biology Division CSIR—Central Drug Research Institute Lucknow Uttar Pradesh India; ^4^ Present address: International Centre for Translational Eye Research (ICTER) Institute of Physical Chemistry (IChF) Marcina Kasprzaka 44/52 01‐224 Warsaw Poland

**Keywords:** COVID‐19 impact, COVID‐19 pandemic, epidemiology, India, vaccines, world

## Abstract

The coronavirus disease (COVID‐19), which the World Health Organization classified as the Sixth Public Health Emergency Of International Concern (PHEIC) on January 30, 2020, is no longer a PHEIC. Millions were affected due to unawareness. The increase in fatalities and shortage of medicine was the first outrage of COVID‐19. As per the Johns Hopkins COVID‐19 resource center database, it was observed that the disease has spread dynamically across 200+ nations worldwide affecting more than 600 million people from 2019 to 2023, and over thousands of people were victimized regularly at a 2% mortality rate (approx.). In the midway, the mutant variants of concern like omicron, and delta have also created havoc and caused significant impact on public health, global economy, and lifestyle. Since 2019, 3 years now passed and the dynamic disease statistics seem decelerated; moreover, the prevalence of COVID‐19 is also fading. The Johns Hopkins resource center has also stopped recording the data of the global pandemic recently from March 10, 2023. Hence, based on the facts, we are presenting a concise report on the pandemic from 2019 to 2023, which includes a brief discussion of the global pandemic. We have highlighted global epidemiology, emphasizing the Indian COVID scenario, vaccination across the globe, and the psychosocial and geopolitical consequences of COVID‐19 with a brief background to pathology, clinical management, and the worldwide response against triage. A lot has changed and still needs to change after three tough years of COVID‐19. Even though science has progressed and advanced research in medicine is pointing toward future generations, there is no standard care supplied for COVID‐19‐like calamities. COVID‐19 cases might have declined but its influence on the society is still stagnant. This COVID experience has taught us that, despite our bleak beginnings, there is always hope for the future and that we must act with foresight to improve things for future generations.

## Graphical abstract

Coronavirus disease‐2019 pandemic has spread across 200+ countries in a very short time span. Many have died and many are getting infected daily. But the rate has slowed down now. Improved pharmacotherapeutics and immunization with precautionary protocols have diminished its impact across the globe. The work focuses on highlighting the disease epidemiology and its socioeconomic, geopolitical, and health impact from 2019 to 2023. (Graphical abstract is created with BioRender.com)

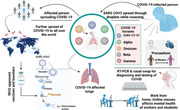

**Abbreviations**
ACE‐2: angiotensin‐converting enzyme‐2
CFR: case fatality rate
COVID‐19: corona virus disease‐2019
ICMR: Indian Council of Medical Research
MERS: Middle East Respiratory Syndrome
MoHFW: Ministry of Health and Family Welfare
PHEIC: Public Health Emergency of International Concern
RNA: ribonucleic acid
SARI: severe acute respiratory infection
SARS: severe acute respiratory syndrome
WHO: World Health Organization

## 1 BACKGROUND

On January 30, 2020, the World Health Organization (WHO) declared the 2019‐nCoV (2019 novel coronavirus) pandemic a Global Public Health Emergency of International Concern (PHEIC). This marked the sixth time such a declaration had been made since 1980. However, during its 15th meeting, the WHO Emergency Committee decided to end the PHEIC status and no longer consider coronavirus disease‐2019 (COVID‐19) a global health emergency [1]. COVID‐19, caused by the zoonotic severe acute respiratory syndrome coronavirus‐2 (SARS‐CoV‐2) virus, has become a global pandemic with a 3.4% mortality rate reported by the WHO on March 3, 2020 [2, 3]. Around 618 million people have been infected with COVID‐19 and over 6.5 million people have perished in past 3 years [4, 5]. After a series of worldwide health emergencies like in 2009—H1N1, 2014—Polio, 2014—Ebola (West Africa), 2016—Zika, and 2019—Ebola (Congo), 2019—COVID was a pounding disaster on the globe [6]. In its early stages, this disease became a pandemic because the general populace was unaware of how deadly and contagious it was. The migration of people from one country to another is the cause of the disease's spread, thus the governments of many nations have implemented tight admission requirements and collected information on the migrants immunization records to stop it. It is more than just a calamity that disease epidemics and economic collapse have spread over the world. During the course of time, variations of the coronavirus have also disseminated globally. The ease with which victims can be identified and tracked down is directly attributable to the effectiveness of treatment and diagnosis in reducing incidence rates. The COVID‐19 pandemic has had a significant impact on various aspects of our lives. Although every calamity has an effect on human life, the effects of the COVID‐19 pandemic will be felt for generations to come. In terms of online education, there has been a distinctive rise in e‐learning, whereby teaching is undertaken remotely and on digital platforms. Moreover, the economic and societal effects of the pandemic have been significant. It has not only caused a physical health crisis but also psychological and mental crises. The pandemic has also had an impact on geopolitical tensions, with the potential to change the global order. The measures taken to prevent the spread of the illness have the potential to exacerbate social, economic, and health‐related disparities which may increase psychological distress for some people. The crisis has led to a dramatic increase in inequality within and across countries. In this overview, we have conceptually compiled the global impact of the pandemic from a variety of publications, newsletters, government websites and analyzed how the globe is currently dealing with this situation.

## 2 MAIN TEXT

### 2.1 SARS‐CoV‐2

The novel beta‐coronavirus (SARS‐CoV‐2) represents the wide domain of coronaviruses belonging to the family Coronaviridae [7], which has severely attacked humanity in the last two decades. These viruses have the potential to induce severe acute respiratory infection (SARI) in healthy individuals [8]. First coronaviral emergence was observed in human coronavirus‐229E in 1965 [9]. In 2002, a similar resurgence of SARS [10] was observed in the Guangdong province of southern China due to SARS‐CoV but was contained rapidly and never reappeared since 2004. Later in 2012, another member of this family, known as the Middle East Respiratory Syndrome (MERS) virus, was discovered in Saudi Arabia [11] caused by MERS‐CoV. These coronaviruses have origin from animal sources. According to reports from the WHO, human transmission of SARS has taken place through direct or indirect contact with infected civet cats. Human transmission of MERS, on the other hand, has taken place from infected dromedary camels; however, there are no strong shreds of evidence supporting human transmission of nCoV‐2. According to the findings that were reported on genetic data, it is presumed that nCoV‐2 has evolved from the bat species [10, 12]. Salespeople in the meat and seafood industry were the patient zero to get the COVID‐19 virus [13, 14, 15]. Since 2019, multiple peaks of the SARS‐CoV‐2 infection have emerged, mainly due to the emergence of new variants, including the Alpha (B.1.1.7), Beta (B.1.351), Gamma (P.1), Epsilon (B.1.427/B.1.429), Delta (B.1.617.2), Mu (B.1.621), and Lambda (C.37), each with a new set of mutations in the viral genome, leading to different pathogenicity, transmissibility, and morbidity [4]. The most recent and rapidly spreading variant of n‐CoV‐2 to appear was the Omicron (B.1.1.529) variation, which the WHO identified on November 26, 2021 [16].

### 2.2 Transmission

Confirmation of cross‐species transmission to humans followed the advent of CoV illnesses with a zoonotic etiology [7, 17]. The transmission of the virus occurs when a healthy individual comes in direct/indirect contact with a COVID‐19‐infected person through handshakes, hugs, kisses, or any sort of physical contact, respiratory droplets from sneezing, and coughing [8]. Additionally, transmission through fomites and surrounding objects when they are exposed/used by an infected person was also confirmed. Besides that, nose, mouth, and eyes via conjunctival contamination are the principal routes of entrance into a healthy person, thus safeguarding these areas is crucial [7, 8, 9, 10, 11]. The virus can live on plastic and stainless steel for up to 72 h, on cardboard for up to 24 h, on copper for up to 4 h, and in the air for up to 3 h [12]. In recent findings, the plausible mode of microdroplet transmission is in the form of aerosols, which can be of very high risk in hospital‐linked and dense demographic locations [12]. Yet, there is no proof that the disease may be spread through the air or by fecal–oral route [12, 13]. These are the steps in the transmission of the COVID was observed across various countries of the globe that led to a pandemic:

**Stage I**: Immigrants from countries where COVID‐19 was prevalent, tested positive for the virus.

**Stage II**: The local transmission of disease from infected those who traveled abroad and came in contact with relatives or acquaintances and all the cases were traced back. (India quarantined itself in this stage to halt community transfer).

**Stage III**: From here, community transmission starts; infection was untraceable.

**Stage IV**: Entry into the epidemic, where it is impossible to stop or prevent the infection and the rise is exponential with increasing deaths. China, Italy, Spain, and the United States are examples of them.

**Stage V**: Emergence of pandemic (spread across 200+ countries).

### 2.3 Indications of COVID‐19

The WHO database indicates that COVID‐19 has variable effects among its sufferers. Children and young people were at low risk as compared to the elder senior citizens (age ≥60 years) and those who have already predisposed to different clinical ailments and comorbidities [14]. The majority of patients showed mild to moderate symptoms and recovered without taking intensive care treatment, but people with underlying health conditions and elder citizens were prone to develop severe diseases and also caused fatalities. The general symptoms shown after 2–14 days of incubation period [15] include high fever, fatigue, sore throat with cough, difficulty in breathing, severe body ache, and very few cases reported of diarrhea, nausea, and a running nose [14]. Later studies revealed the symptoms like loss of smell and taste [13], and pink eye [18] associated with it. The National Health Commission of the People's Republic of China released a special report in March 2021 detailing the identification of 1541 patients who tested positive for COVID‐19 but displayed none of the aforementioned symptoms and instead served as carriers of the virus [18]. In contrast, pathological examinations of critically and fatally ill patients indicated dramatically decreased numbers of lymphocytes, T cell subsets, and eosinophils. When compared to survivors, nonsurvivors either had stable or increased neutrophil levels, whereas neutrophil counts, interleukin‐6, procalcitonin, serum amyloid A protein, and C‐reactive protein levels remained steady or decreased in survivors [19]. Meanwhile, the majority of COVID‐19 patients with severe or fatal outcomes were seniors, especially those with pre‐existing chronic illnesses. Age and underlying illnesses reduce immunity, making life more challenging for the aged, which can delay or even prevent infection clearance. Thus, the elderly are a crucial demographic for preventing the spread of COVID‐19 [20].

### 2.4 Precautions and prevention

In an effort to educate the public and prevent the further spread of COVID‐19 and its variations, WHO has issued multiple guidelines and launched online courses [21, 22]. Distancing oneself socially is the first line of defense against the spread of disease. According to the Indian Council of Medical Research (ICMR) stated research, a COVID‐19‐infected patient can infect 406 others if the patient does not practice social distancing for 30 days [23]. Other possible ways to stop the spread are to avoid direct or indirect physical contact, maintain good sanitary practices such as frequently wearing a mask, handwashing with soap or sanitizers, covering nose and mouth with a handkerchief/tissue while sneezing and coughing, avoid touching eyes–nose–mouth frequently, staying indoors to minimize public gatherings, and self‐contamination [24]. Regular bathing with antiseptic soap and clean running water, ideally twice daily (morning and night), as well as frequent washing of clothing with detergents [25]. Safeguarding personal care by boosting host immunity should be our primary concern, doing regular exercise, yoga, and consuming fruits and vegetables enriched with Vitamin C and E, intake of warm water, and steam inhalation will be beneficial for respiratory health [26].

### 2.5 Diagnostic tools and tracing strategies

**Figure 1:**
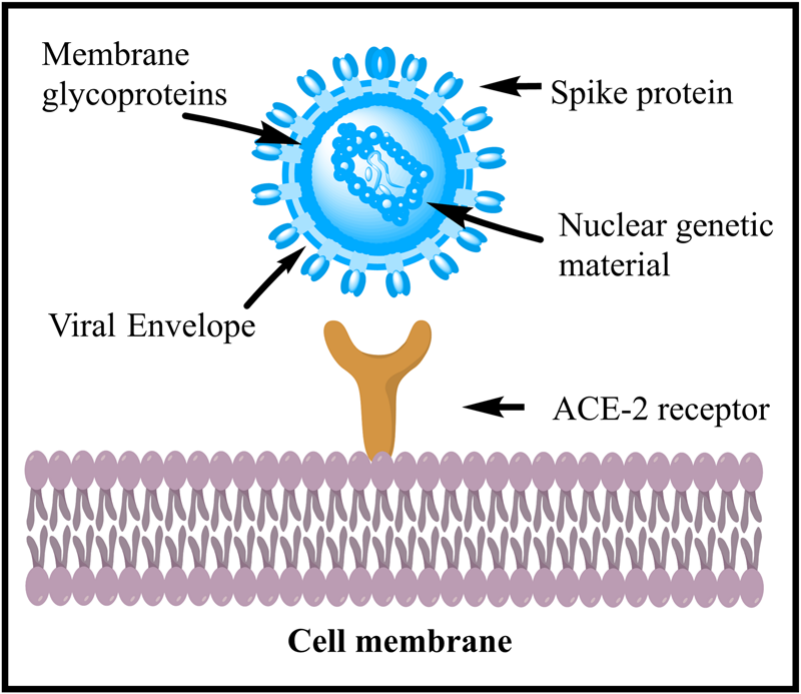
Diagrammatic illustration representing the binding of novel coronavirus (nCoV) viral spikes onto angiotensin‐converting enzyme‐2 (ACE‐2) cellular receptor and its target for intracellular entry [29, 30, 31, 32, 33, 34].

The glycoprotein enveloped spike (S) structure of nCoV‐2 was discovered through molecular biology investigations and was of paramount importance in the production of therapeutic biological products such as diagnostic tests, vaccinations, and antibodies [27, 28]. N‐CoV‐2 has a positive single‐stranded RNA virus with a genome length of 30 kb, which is the largest among RNA viruses [29, 30]. Its trimeric spikes [31] have a strong affinity toward the angiotensin‐converting enzyme‐2 receptor [32]; after binding, it enters into the host cell, and using host machinery, it replicates itself and compromises target immunity [33, 34] as illustrated in Figure 1. For corona‐positive suspects with symptomatic/asymptomatic COVID‐19, the WHO and the US Food and Drug Administration recommend using a nucleic acid amplification test such as real‐time reverse transcriptase‐polymerase chain reaction (RT‐PCR) [35, 36] and serological antigen (Ag)–antibody‐based assays [36]. SARS‐CoV‐2 protein Ag detection immunoassays are frequently used for diagnosis. Diagnostic results from lateral flow assays can be obtained in about 15 min, making them the gold standard for usage at the point of care [37]. It is evident that early therapy with neutralizing monoclonal antibodies is good for ambulatory patients. Its success in hospitalized COVID‐19 patients has been variable, despite the substantial risk of catastrophic disease progression in people with mild or moderate disease [38]. Initially, chest X‐ray cum peritoneum computed tomography scan imaging techniques and whole viral genomic RNA identification were used as diagnostic tools to find the viral genome in infected patients [39, 40]. Consequently, the evaluation of chest radiograph (CXR) was a subsequent diagnostic screening technique taken into consideration alongside RT‐PCR [11, 41]. Portable devices can considerably reduce the danger of virus transmission, which made CXRs the favored method of screening during the pandemic; however, the shortage of competent radiologists to interpret imaging data was a significant barrier to CXR screening at the time [42]. A recent virology investigation found that the reverse transcription‐loop‐mediated isothermal amplification assay was an effective diagnostic tool for use in healthcare personnel [43].

### 2.6 Clinical management and pharmacotherapeutic treatment

The spread of disease can be halted by taking protective and preventative measures [44, 45]. Patients with a confirmed case of COVID‐19 were treated according to their symptom severity, which was categorized as either mild, severe, or critical. In severe and critical cases, SARI is triggered by acute respiratory distress syndrome, organ dysfunction, and septic shocks with critical cardiac and kidney damage [46]. In a report, WHO mentioned that elderly people in the age group of >60 years can easily get the disease, and the mortality rate among them is also higher as compared to youngsters and children [47]; very rare cases are reported regarding the death of pediatric patients but they could have served as carriers [48]. Transmission of the virus through the amniotic fluid and breastfed milk of pregnant women was found negative and the fetus also tested negative [8, 49]. Therapeutic options include the combination of lopinavir and ritonavir (400/100 mg every 12 h), chloroquine (500 mg bis a day), and hydroxychloroquine (200 mg bis a day) with α‐interferon (5 million units by inhalational aerosol bis a day) as one of the regimens for the treatment [50]. In an open‐label nonrandomized clinical trial, it was reported that azithromycin and hydroxychloroquine were very effective for the treatment of COVID‐19; reduction in pulmonary viral load was increased by the use of azithromycin [51]. Additionally, therapeutic repurposing of medicines with potential against COVID‐19 and its novel variants have also been cited in numerous papers as being successful, for example, nirmatrelvir [52], baricitinib [53], lopinavir [54], ritonavir [54, 55], darunavir [56], favilavir, favipiravir [54, 57], remdesivir [58, 59, 60], ribavirin [39, 58, 61], galidesivir [40], arbidol [56], nitazoxanide [58], and ivermectin [62]. Other nutraceuticals that played an important role include hesperidin and Patanjali's Divya Swasari Coronil Kit for the management of COVID‐19.

### 2.7 Post‐COVID syndrome

The Department of Health and Human Services, in collaboration with the Center for Disease Control, has introduced the term “Post‐COVID Conditions” (PCCs). This term refers to a phenomenon where individuals who have been infected with COVID‐19 may continue to experience clinical symptoms for more than 4 weeks after the initial infection. Other names used to describe this condition include long COVID, long‐haul COVID, postacute COVID‐19, persistent post‐COVID syndrome, and chronic COVID. Common clinical symptoms associated with PCCs include cough, fever, irregular breathing, fatigue or tiredness, headache, dizziness, persistent loss of taste or smell, depression or anxiety, and muscle and joint pain, among others [63, 64].

### 2.8 COVID‐19 vaccinations

**Figure 2:**
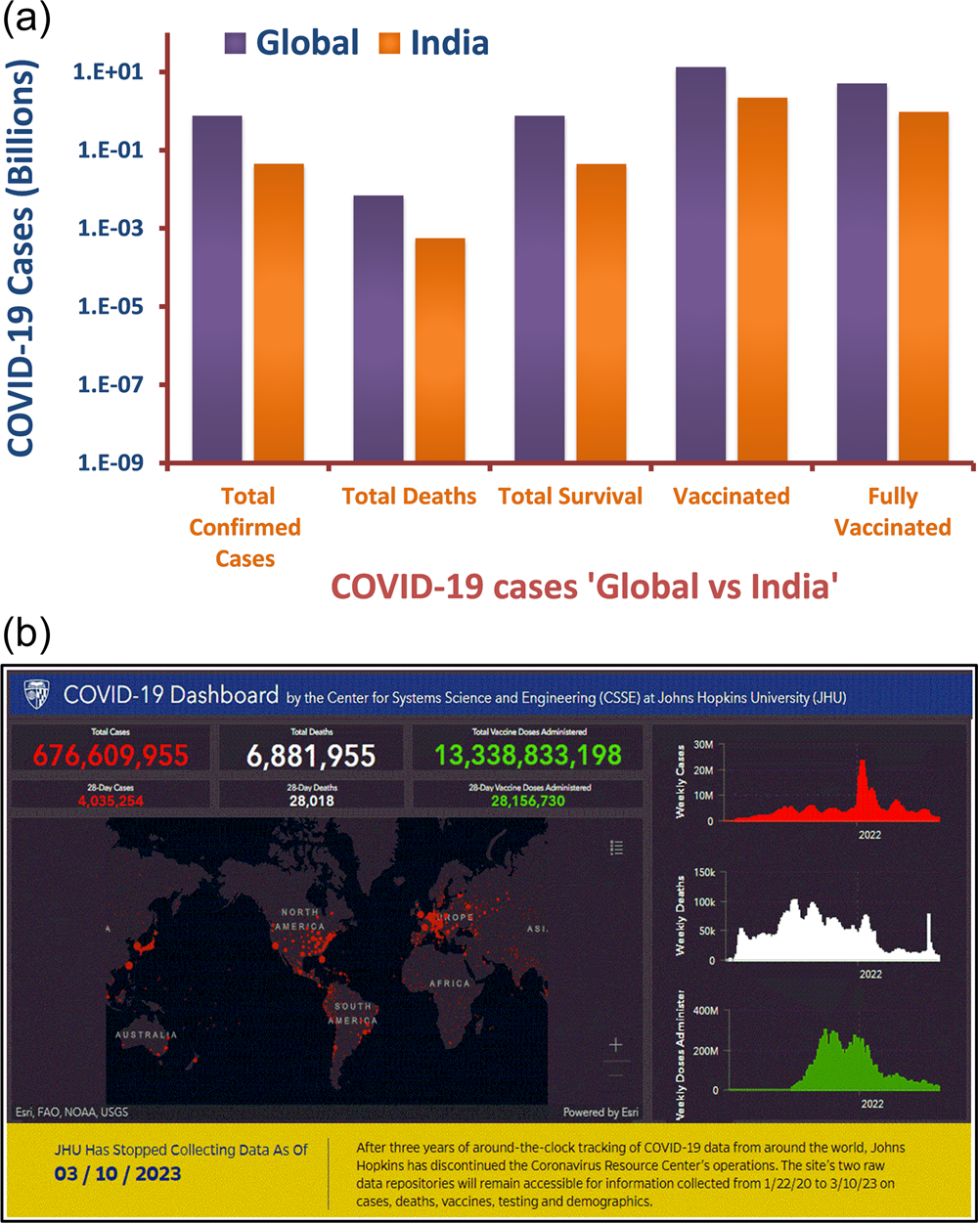
(a) The bar graph represents comparative the cumulative numbers of COVID‐19 cases, deaths, recoveries, and immunization stats across the globe and in India. (b) The COVID‐19 dashboard from John Hopkins University showing data as of March 10, 2023 [5].

COVID‐19 vaccination has substantially altered the course of the pandemic, saving tens of millions of lives globally [65]. The WHO published a statement in September 2022 reporting that 172 vaccines were in clinical development and another 199 were in preclinical testing. There were numerous pharmaceutical companies racing to develop a vaccine for COVID‐19 before it was approved by the WHO to curb the pandemic including Pfizer, AstraZeneca, Johnson & Johnson, Bharat Biotech, Sinovac, and Moderna described in Table 1. These vaccines are designed using protein fragments, RNA, a nonreplicating viral vector, and inactivated viruses as their basis. Some countries now compel tourists, students, and migrant workers to show immunization documentation as a result of the vaccines' widespread impact. Free COVID‐19 vaccination was the first step in what is being called the world's largest immunization program, and on January 16, 2021, the government of India asked all of its citizens to take part [44]. As per the data from daily media reports, India has supplied COVID‐19 vaccines to many countries, including its neighbors such as Bhutan, Maldives, Nepal, Myanmar, and Bangladesh. India has shipped millions of COVID vaccine doses around the world. The Indian government has supplied over 22.9 million doses of COVID‐19 vaccines to 25 countries on a commercial basis to date, and more are on the way. The vaccines supplied by India include Covishield (Oxford‐AstraZeneca vaccine manufactured by Serum Institute of India), Covaxin (manufactured by Bharat Biotech), Sputnik V, and NVX‐CoV2373 (Novavax) [66]. Modern science has also developed vaccines for administration via the nasal route, and many countries (including India and China) have begun to embrace this alternative to the intravenous approach [67]. Two other mucosal COVID‐19 vaccines have been developed, although data on their effectiveness is scarce. More than 5000 doses of a COVID‐19 nasal spray vaccine made by the Razi Vaccine and Serum Research Institute in Karaj were distributed to the public since the vaccine was licensed in Iran in October 2021. Meanwhile, Russian health ministry has approved an intranasal spray version of Sputnik V, the modified version of injectable COVID‐19 vaccine [68]. The daily vaccination data is being updated by the Indian Ministry of Health and Family Welfare (MoHFW); as of March 23, 2023, the total number of Indians who have been vaccinated is 2,206,528,710 (Figure 2a) [69]. Adverse effects in vaccines were reported including drowsiness, muscular pain, headache, chills, joint pain, fever, and skin problems like pernio/chilblains, shingles, and herpes simplex flares explained in Table 1 [70]. However, inadequate access to vaccines in low‐income countries has limited the impact in these settings, reinforcing the need for global vaccine equity and coverage [65].

**Table 1:** List of WHO‐approved vaccines for immunization against the COVID‐19 pandemic highlighting the mechanism of action with reported adverse reactions.

Pharmaceutical manufacturer	Name of vaccine	Mechanistic action	Adverse drug reaction	Reference
Pfizer‐BioNTech	BNT162b2	For stability, deliver mRNA encoding the entire S protein with two equal proline substitutions. An equal amino acid substitution is one that exchanges one for another biochemically related amino acid	Fatigue, headaches, muscular pains, chills, and fever are the common side effects, while pain at the injection site causes discomfort, redness, and deem	[71, 72, 73]
Moderna	mRNA‐1273	For stability, deliver mRNA encoding the entire S protein with two equal proline substitutions. An equal amino acid substitution is one that exchanges one for another biochemically related amino acid	Fatigue, headaches, muscular pains, chills, and fever are common side effects, while pain at the injection site causes discomfort, redness, and edema	[71, 72, 73]
Janssen (Johnson & Johnson)	Ad26.COV2.S	Passive immunity against SARS‐CoV‐2	Post side effects are fatigue, fever, headache, soreness at the injection site, nausea, and diarrhea are some of the side effects	[74, 75]
AstraZeneca	AZD1222	The S protein produced by the mRNA vaccines above is processed and presented by the MHC I and MHC II complexes by nonimmune and immune cells. Immunological antigen‐presenting cells	Fatigue, headaches, muscular pains, chills, and fever are common side effects, while pain at the injection site causes discomfort, redness, and edema	[73, 76]
Sinovac	CoronaVac	Inactivated virus	Post side effects are fatigue, fever, headache, soreness at the injection site, nausea, and diarrhea are some of the side effects	[75, 77]
Sputnik V and Sputnik Light	Gamalia Institute of Epidemiology and Microbiology Moscow, Russia	To create antibodies against S proteins	Post side effects are fatigue, fever, headache, soreness at the injection site, nausea, and diarrhea are some of the side effects	[75, 78]
Covishield	Serum Institute of India Pvt. Ltd.	SARS‐CoV‐2 S glycoprotein is expressed locally, eliciting neutralizing antibody and cellular immunological responses	Mild side effects include dizziness, headache, discomfort, muscular spasms, myalgia, and paresthesia	[79, 80]

Abbreviations: COVID‐19, coronavirus disease 2019; mRNA, messenger RNA; SARS‐CoV‐2, severe acute respiratory syndrome coronavirus‐2; WHO, World Health Organization.

## 3 GLOBAL EPIDEMIOLOGY

**Figure 3:**
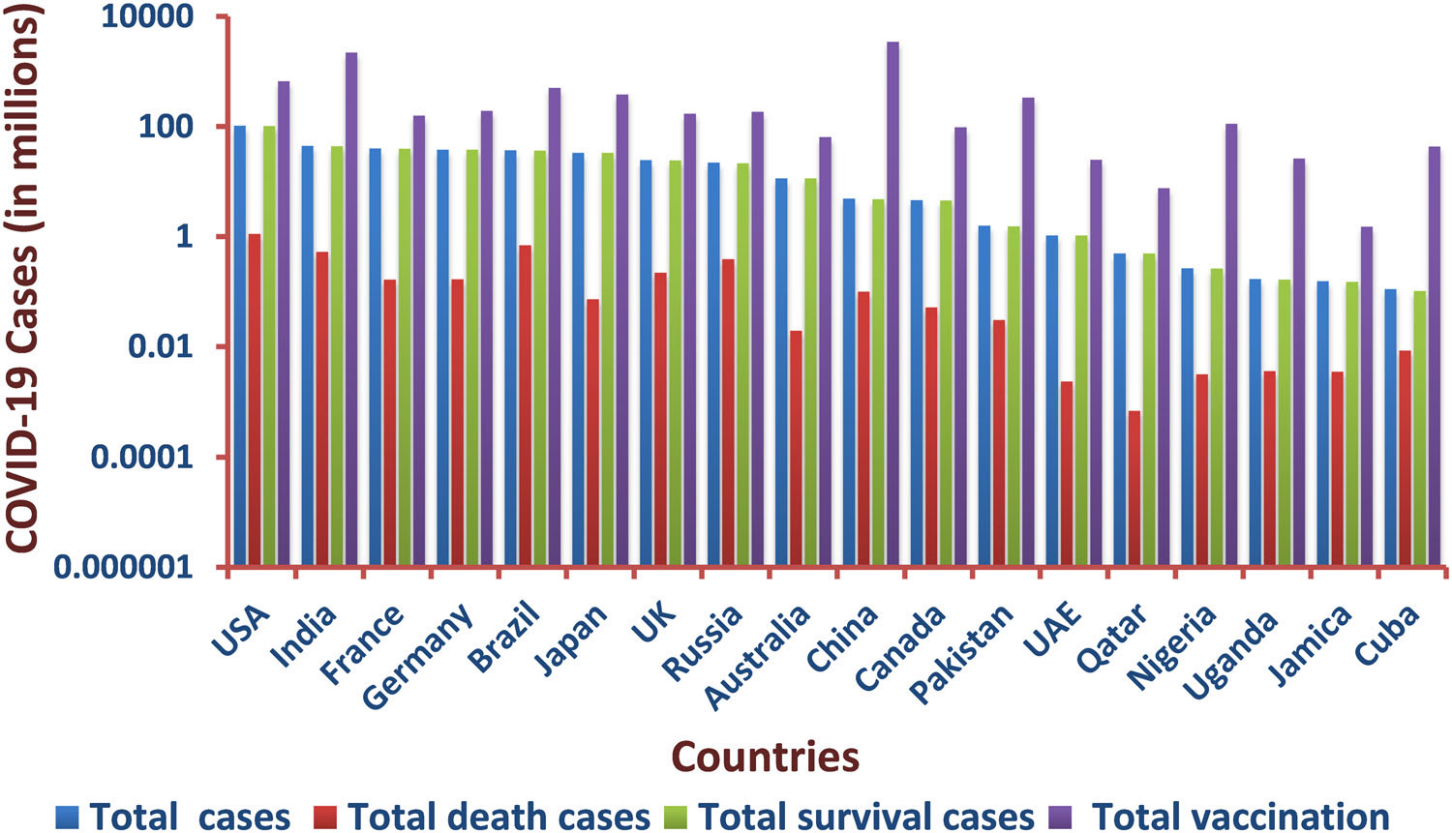
The graph represents statistical data of total cases, deaths, and survival particulars of countries that were severely affected by coronavirus disease 2019 (COVID‐19) worldwide.

COVID‐19 has rapidly expanded across the globe since it was first emerged, with over 550 million confirmed cases and over 6 million deaths documented across approximately 200 nations in just 3 years [5]. The WHO coronavirus disease situation dashboard provides a central location for further information and provides official daily counts of COVID‐19 cases and deaths globally, as well as vaccination rates and other immunization statistics. A huge amount of work has been contributed by the Johns Hopkins University (JHU) database in reporting corona‐positive patients, total cases, death cases, and also vaccination status, which is growing rapidly every day as countries try to slow the spread of the pandemic. However, the international COVID‐19 data repository (JHU) has been shut down after 3 years of operation because the severity of the condition seems to be no longer a concern. According to recent reports, the number of average daily cases of COVID‐19 is decreasing, and the mean mortality rate has decreased from 3.4% to 2% around the world. The conclusive data were published by JHU on March 10, 2023, and it was reported around the globe as follows: there were a total of 676,605,955 cases, 6,881,955 deaths, and 6,669,728,000 total vaccines performed on 13,338,833,198 individuals as shown in Figure 2b [81]. Europe has had 273,666,626 confirmed cases and 2,136,714 deaths after three years. According to the WHO, as of March 9, 2023, the United States will have seen the most deaths of any continent (Live COVID‐19 Information Center) [5, 81]. Although African countries were largely untouched by the COVID‐19 epidemic, they remain at risk due to inadequate health care and potentially risky trade relations with China, Italy, and other countries hit hard by the pandemic [82]. The global cases of confirmed COVID‐19 surpassed 0.7 billion and increasing daily [81]; new variants spread undetected due to a shortage of laboratories capable of handling COVID‐19 samples, as well as a lack of financing or restrictions on importing reagents and equipment [83]. Although the United States possesses top‐notch medical facilities and conducts roughly 100,000 tests every day, the initial phase of the COVID‐19 outbreak was made much worse due to the absence of immunizations, illness‐specific medications, effective disease intervention tactics, and proactive government policies. Since April 2020, other countries including Iran, Belgium, and Germany have begun to flatten the curve [5, 81]. Maximum daily deaths have been documented in China, Italy, and France; nevertheless, a study found that the recovery rate was very similar for Italy and China, while the transmission rate and associated death toll varied widely by culture and lifestyle [50]. The number of newly reported positive cases each day has been falling in countries like China and South Korea, although cases of reinfection in previously negative patients who have since made a full recovery have been documented. According to data issued by the Korea Centers for Disease Control and Prevention, the virus is reactivated in 51 of 7000 individuals who had previously shown complete recovery which was a very alarming call [51]. Lack of early COVID‐19 containment strategy and action plan, poor testing facilities, lack of treatment options, scarcity of life‐supporting systems in hospitals, and lack of information against COVID‐19 are responsible factors for the rapid global spread of the disease. There have been more deaths in the United States than in any other country since the COVID‐19 pandemic struck 3 years ago. This failure is attributable to the fact that neither the United States nor European countries have implemented even the most fundamental epidemic containment plan. In contrast, the epidemic was contained in Turkey and Germany with relatively low fatality rates [53]. In Germany, COVID‐19 emerged in the younger and sporty population who transmitted the disease in their professional network, which was easily traced and showed milder symptoms with insignificant casualties under good governance policies and nationwide lockdown [84]. In Turkey, a strict curfew was imposed under the age of 20 and above 65 with free distribution of masks and protective gears to contain the COVID‐19 spread [85, 86]. Sweden, after a huge number of deaths, employs the concept of “herd immunity” to tackle the epidemic [87], but it seems to be ineffective as epidemic progression is constant. The global demographic data related to COVID‐19 is graphically presented in Figures 2 and 3, respectively.

## 4 INDIAN EPIDEMIOLOGY

**Figure 4:**
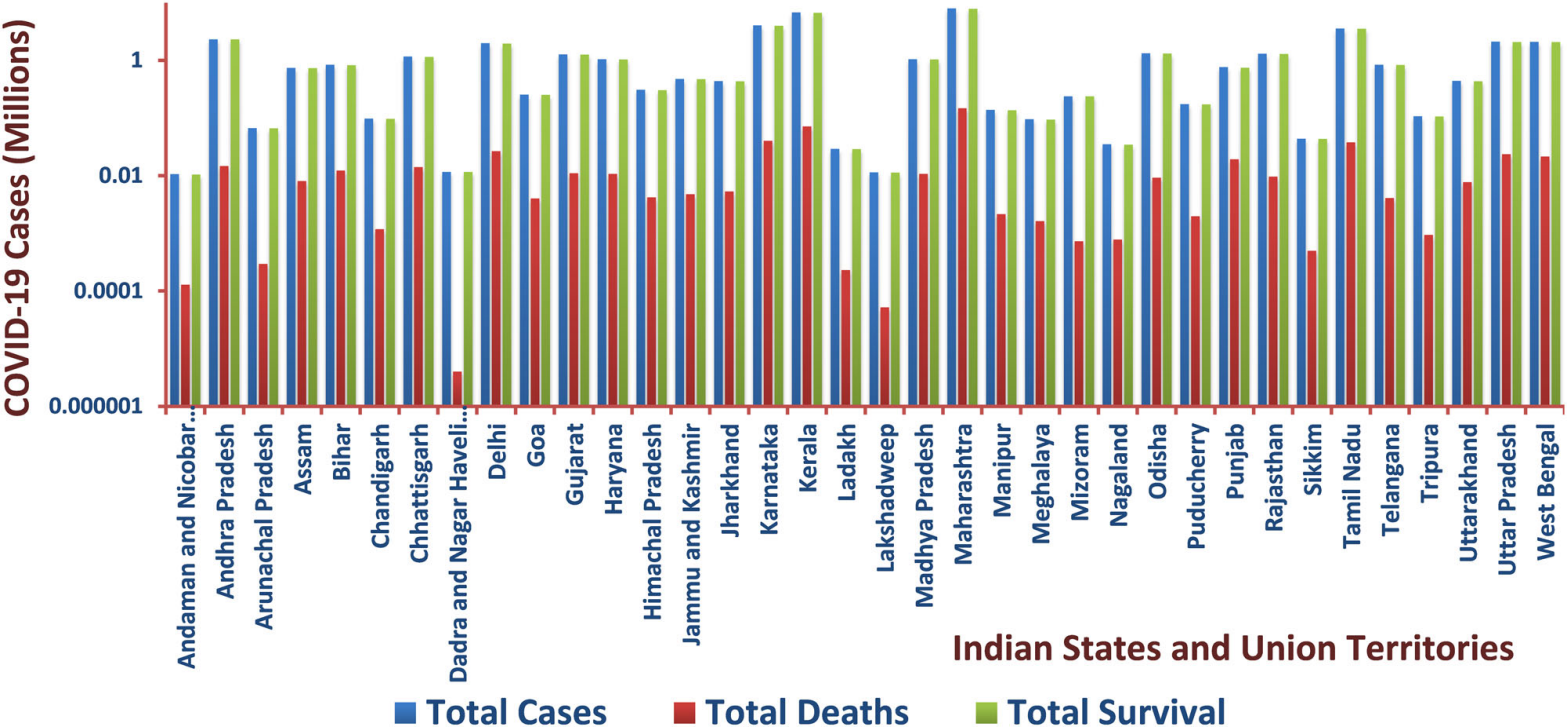
The bar graph represents statistical numerals of total cases, deaths, and survival particulars of Indian states and union territories, which were severely affected by the pandemic.

India was on the cusp of experiencing the serious trial brought on by the pandemic's overall effect on developing nations. On January 30, 2020, in the state of Kerala, the first corona‐positive case was reported in the nation with over 1.40 billion people. One of the major causes of the spread of disease in India was a lack of preparation and a shortage of hospital beds and oxygen facilities. Confirmed cases of COVID‐19 rose sharply after the virus emerged, with the maximum density found in Maharashtra State, followed by Delhi, Madhya Pradesh, Tamil Nadu, Rajasthan, and finally the rest of India, where the %CFR was assessed to be somewhere around 3.3%. Kerala has the least case fatality rate (CFR), whereas Punjab has the most among the declared hotspots in the nation (considering the infected population of >100). While in Kerala overall number of active cases was the highest for a month, nine other states have since surpassed it. Kerala's success in reducing the regional epidemic curve can be attributed to the state's decentralized and three‐tier healthcare system, as well as route map contact tracking and testing. Moreover, the infected were mostly middle‐aged (20–50 years), which has reduced its CFR to 0.76%. These interventions have aided Kerala in reducing the number of COVID‐19 cases in the state [88, 89]. However, lockdowns and curfews have been implemented across the country, and as a result, the number of incidents has dropped significantly. The Indian government took preventative measures, including rapid testing of suspected patients and contacts, contact tracing, and the construction of quarantined treatment facilities stocked with personal protective equipment for healthcare employees. In such a dire situation, the group of authorized employees in each city and district of the nation provided seamless door‐to‐door contactless service to meet the demand and supply of essential goods, medicines, and services. Therefore, states where mortality rates were particularly high have seen their mortality ratios drop and their survivor rates rise. On March 23, 2023, instances were recorded in the states of Maharashtra (379), Delhi (292), Madhya Pradesh (35), Tamil Nadu (480), Rajasthan (163), and Kerala (2007), according to the COVID‐19 portal maintained by the MoHFW of the Government of India. Figure 4 displays the declining mortality rate in India, which was 1.17%, and the high number of discharge cases, which was 98.80%. As per the latest data accessed on March 23, 2023, the overall number of patients discharged was 44,160,997; the total number of deaths was 530,816; and the total number of immunized individuals was 2,206,528,710 (including both dose 1 and dose 2 vaccinations) [69].

## 5 NAVIGATING THE PANDEMIC: IMPACTS AND INTERVENTIONS

If we look back in time, we can see that every time a pandemic catastrophe occurs, it results in widespread destruction. The unthinkable COVID‐19 epidemic exemplified a worldwide public emergency that threatened the lives of people everywhere. COVID‐19 has no bias in its neutralization of the majority of nations (particularly superpowers), individuals of varied social, economic, cultural, and economic backgrounds, rich and poor alike. The worldwide fight against COVID‐19 has exposed a state of hopelessness and vulnerability.

### 5.1 Impact of COVID‐19 on the global healthcare management

**Figure 5:**
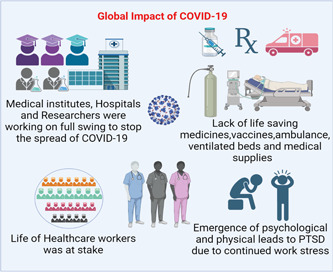
Global impact of coronavirus disease 2019 (COVID‐19) on the healthcare field including doctors, hospitals, researchers, and medical institutions. PTSD, post‐traumatic stress disorder.(Created with BioRender.com)

The COVID‐19 pandemic has presented unprecedented challenges to the healthcare field, affecting global medical institutions, healthcare professionals, patients, hospitals, healthcare researchers, and the prevention and treatment of COVID‐19 (Figure 5). We have critically analyzed the multifaceted impact of the pandemic, exploring the implications on healthcare systems, the challenges faced by various players, and the strategies implemented for prevention and treatment. Understanding these dynamics is crucial for enhancing future pandemic preparedness and improving healthcare response strategies [90].

#### 5.1.1 Impact on global medical institutions and hospitals

The COVID‐19 pandemic has placed immense strain on global medical institutions, revealing vulnerabilities and necessitating swift adaptations. Hospitals and healthcare facilities have faced overwhelming patient surges, leading to overcrowding, and shortages of critical resources like beds, ventilators, PPE, and medical supplies. Weaknesses in healthcare infrastructure, particularly in resource‐limited settings, have been exposed, highlighting the need for robust and resilient systems. Rapid reorganization and reprioritization of healthcare services by hospitals have often resulted in delayed or deferred care for non‐COVID‐19 patients, including those with severe comorbidities [91, 92]. COVID‐19 is posing catastrophic financial issues for international hospitals and healthcare facilities. The American Hospital Association forecasts that America's hospitals and healthcare systems will lose $202.6 billion in revenue or $50.7 billion every month on average. Furthermore, providing an effective healthcare response to COVID‐19 might cost low‐ and middle‐income countries US$52 billion (equal to $8.60 per person) every 4 weeks [93]. The economic impact on medical institutions has been substantial, with decreased revenues, increased costs, and disrupted healthcare delivery, exacerbating existing disparities in access to quality healthcare. Long‐term sustainability and adaptability are now key considerations for future crises [94, 95, 96].

#### 5.1.2 Challenges faced by healthcare professionals

Healthcare professionals have faced numerous challenges during the COVID‐19 pandemic. Physical strain includes increased workloads, prolonged shifts, high exposure risks, and shortages of protective gear and medicines, leading to increased infection rates among healthcare workers and the unfortunate loss of lives. The psychological impact on healthcare professionals has been significant, with elevated levels of stress, anxiety, burnout, moral distress, and posttraumatic stress disorder. Witnessing the suffering and loss of patients, working in high‐pressure environments, and making difficult ethical decisions have taken a toll on their well‐being. A study of the different demographics of suicide cases found that the risk ranged from 2.73 in physicians to 0.44 in architects and engineers [97]. Easy access to means of suicide is a key risk factor when considering the risk of suicide. According to a survey, healthcare workers, such as nurses, doctors, and chemists, are more likely to commit suicide using poisons [98]. The COVID‐19 outbreak has greatly increased tension, anxiety, depression, and insomnia among healthcare professionals, according to studies. Adequate support systems, mental health services, and recognition of their contributions are essential for preserving the well‐being of healthcare professionals [94, 95, 96]. Some essential components may include support and wellness programs for all healthcare staff, lifestyle and mental health exams, and appropriate referrals for help or treatment [99]. In the face of an emergency, it can be arduous to look beyond the immediate crisis. Nevertheless, considering the profound and lasting effects of the ongoing pandemic, we strongly urge the healthcare community to prioritize the mental health needs of healthcare workers both in the present and the future. The continuous stressors stemming from the pandemic are anticipated to exacerbate mental health concerns, which may even include an increased risk of suicide. Therefore, it is imperative that healthcare professionals take proactive measures to address these issues and provide the necessary support to safeguard the well‐being of those on the front lines. By acknowledging and addressing the mental health challenges faced by healthcare workers, we can ensure their resilience and promote a healthier and more sustainable healthcare system.

#### 5.1.3 Challenges faced by patients

COVID‐19 has posed unique challenges for patients, both physically and psychologically. Beyond those directly affected by the virus, individuals requiring routine healthcare services, elective procedures, and non‐COVID‐19 emergencies have faced disruptions due to resource reallocation and fear of infection. Delayed or deferred care has resulted in adverse health outcomes, exacerbating existing health conditions and leading to potentially avoidable complications. The psychological impact on patients has been profound, with increased anxiety, depression, isolation, grief, and disrupted social support networks. Moreover, there is a substantial need for healthcare providers and policymakers to recognize the potential risks faced by females such as polycystic ovary syndrome patients and to ensure their adequate protection and support during the pandemic [100, 101, 102]. Other diseases like mucormycosis and keratomycosis fungal infection that has gained attention during the COVID‐19 pandemic. These are the secondary comorbidities that emerged during the pandemic in patients. Prevention and management of these fungal infections involve a multidisciplinary approach, including the involvement of infectious disease specialists, ophthalmologists, otolaryngologists, and other healthcare professionals. Timely diagnosis, appropriate antifungal treatment, and surgical interventions, if necessary, are essential for improving patient outcomes [103, 104, 105]. Vulnerable populations, such as the elderly, individuals with pre‐existing mental health conditions, and marginalized communities, have been particularly affected. Strategies to ensure continuity of care, telehealth services, and comprehensive mental health support are crucial to addressing the needs of patients during and beyond the pandemic [106, 107, 108, 109, 110].

#### 5.1.4 Impact on healthcare research & development and life science researchers

The COVID‐19 pandemic has disrupted ongoing healthcare research projects worldwide. Lockdown measures, travel restrictions, and the redirection of resources have resulted in delays, interruptions, and, in some cases, complete termination of studies. Clinical trials, observational studies, and basic science research have been particularly affected, impacting the timely generation of critical data and hindering the progress of medical research. Moreover, research facilities and laboratories have faced limitations in capacity due to physical distancing requirements and reduced access to necessary equipment and supplies. These disruptions have created a significant setback for ongoing projects and have necessitated adaptive strategies to resume and complete research studies [111, 112, 113, 114, 115].

The emergence of the COVID‐19 pandemic has led to a rapid shift in research priorities within the healthcare field. Resources, funding, and scientific attention have been redirected toward understanding the virus and developing diagnostic tools, therapeutic interventions, and vaccines [116, 117]. Studies focused on other diseases and conditions have been deprioritized or repurposed to address the pressing needs of the pandemic. This shift has both short‐ and long‐term consequences, as it impacts the progress made in various areas of healthcare research. Additionally, the pandemic has highlighted the importance of interdisciplinary research and collaboration, as researchers from diverse fields have come together to address the urgent challenges posed by COVID‐19 [118, 119, 120, 121, 122].

Life science researchers have encountered numerous challenges during the COVID‐19 pandemic. Laboratory closures, limited access to research facilities, and disruptions to supply chains have impeded experimental work and data collection. The need for physical distancing and remote work arrangements has altered collaborative dynamics and hindered the exchange of ideas and knowledge sharing. Additionally, researchers have faced increased pressure to deliver timely results and contribute to the global efforts against COVID‐19. These challenges have not only impacted the productivity and progress of individual researchers but have also affected their mental well‐being, as they grapple with uncertainties and the need to adapt to rapidly evolving circumstances [123, 124, 125].

Despite the challenges, the COVID‐19 pandemic has sparked unprecedented collaboration among researchers globally. Scientific communities and institutions have come together to share data, research findings, and resources. Open science initiatives have gained traction, enabling rapid dissemination of research outputs and facilitating collaborations. The sharing of genetic sequences, clinical data, and treatment protocols has accelerated the understanding of the virus and informed the development of diagnostics, therapeutics, and vaccines. Collaborative efforts have transcended geographical boundaries and disciplinary silos, fostering synergies and enabling scientific breakthroughs at an unprecedented pace [126, 127, 128, 129].

The COVID‐19 pandemic has generated a wealth of new medical knowledge and scientific discoveries. The rapid development of diagnostic tests, the identification of potential therapeutic targets, and the successful development and deployment of vaccines highlight the remarkable progress made in a relatively short period. Researchers have gained insights into the pathogenesis of the virus, host immune responses, and risk factors for severe disease outcomes. Additionally, the pandemic has accelerated the adoption of innovative research methodologies, such as virtual collaboration and remote data collection. With restrictions on in‐person interactions and travel, researchers and scientists have increasingly turned to virtual platforms and digital tools to continue their work and collaborate with colleagues around the world. [130, 131].

#### 5.1.5 Impact of COVID‐19 on the number of outpatient visits and hospitalizations in hospitals

The COVID‐19 pandemic has had a significant impact on the number of outpatient visits and hospitalizations in hospitals worldwide. Here is a summary of the key effects:

a. *Decrease in outpatient visits*: Many hospitals experienced a significant decline in outpatient visits during the pandemic. This decline can be attributed to various factors, including fear of contracting the virus, lockdown measures, and prioritization of COVID‐19 care. People with nonurgent or nonemergency medical conditions often chose to delay or avoid seeking medical care, leading to a decrease in outpatient visits [132].
b. *Postponement of elective procedures*: To free up resources and prioritize COVID‐19 patients, many hospitals postponed elective procedures and surgeries. These procedures, such as cosmetic surgeries or nonlife‐threatening surgeries, were rescheduled to reduce the risk of COVID‐19 transmission and conserve medical supplies. As a result, the number of hospitalizations for elective procedures decreased significantly [132].
c. *Increased hospitalizations for COVID‐19*: Hospitals experienced a surge in hospitalizations due to COVID‐19 patients. These patients required intensive care, respiratory support, and monitoring, leading to an increased strain on hospital resources. As a result, available beds, equipment, and healthcare staff were often redirected to treat COVID‐19 cases, limiting the capacity for other non‐COVID‐19 hospitalizations [133].
d. *Fluctuations in hospitalizations*: The number of hospitalizations during the pandemic has fluctuated based on the severity of outbreaks in different regions and the implementation of control measures. As infection rates rose and fell, hospitals faced surges in admissions during peak periods and relatively lower occupancy during lulls. These fluctuations placed additional strain on hospital resources and required adaptive management strategies [132].
e. *Long‐term health consequences*: COVID‐19 itself can lead to severe illness and long‐term health complications. Some patients who contracted the virus experienced ongoing symptoms and required prolonged hospitalization or outpatient care. This added burden on healthcare systems, as managing post‐COVID‐19 conditions became an important aspect of hospital care [132, 134].

It is important to note that the impact of COVID‐19 on outpatient visits and hospitalizations varied across regions and over time, depending on the severity of the pandemic, healthcare system capacity, and public health responses. The scenario is well explained in the study conducted by Ping Wang and colleagues examined the impact of COVID‐19 on outpatient numbers across multiple departments in a general hospital in Beijing. They found an overall decrease in outpatient numbers in 2020 compared to 2019, with variations observed among different departments. The study highlighted the importance of patient reservation rates and recommended the classification of departments based on changes in outpatient numbers and reservation rates for effective resource allocation [135].

The results of the study indicated that there was an overall decrease of 33.36% in outpatient numbers in 2020 compared to 2019. Among the departments, 10 experienced a decrease in outpatient numbers by more than 33.36%, while two departments actually saw an increase. This suggests that the impact of COVID‐19 on outpatient visits varied across different departments. The researchers also examined a previously unexplored indicator, which was the monthly departmental patient reservation rates. In 2020, the overall patient reservation rate for the 24 departments was 82.22% of the 2019 reservation rate. Among the departments, 14 had reservation rates below 82.22%. This indicates that patient reservation rates also varied across departments and different months. The study found that the impact of COVID‐19 on different departments was not uniform. It suggested that well‐known departments and those related to tumor treatment might be less affected by the pandemic and could even experience an increase in patient numbers. This implies that certain departments may have been better able to adapt to the challenges posed by COVID‐19.

The researchers emphasized the importance of patient reservation rates as an indicator that should be given attention. This implies that monitoring and analyzing patient reservation rates can provide valuable insights into the impact of COVID‐19 on outpatient numbers. Based on their findings, the researchers recommended that hospital managers classify departments based on changes in outpatient numbers and patient reservation rates. This classification can help in allocating outpatient doctor resources more effectively during the pandemic. The researchers also proposed the adoption of accurate, dynamic, and humanized management strategies to respond to the changing circumstances caused by COVID‐19 [135, 136].

### 5.2 Economic impact

Since the 2008–2009 financial tragedies, World Trade Organization and Organization for Economic Co‐operation and Development have indicated COVID‐19 as the next emerging global economic crisis and the era of the worst recession in history since the 1930s. The United States and the United Kingdom, two of the world's largest economies, are shrinking every day; worldwide trade fell by 13%–32% in 2020 due to COVID‐19 and is still falling [137]. It is true that some industries thrived during the lockdown era, like online entertainment and broadcasting, the telecommunications industry, and the pharmaceuticals and diagnostics sector. However, others, such as banking, automobiles, textiles, tourism and aviation, IT, electronics, shipping, and solar power, will have a much harder time making it [138]. Yet, several countries' economies have already begun to slow. The Indian economy also suffered the same fate, its severity determined by the pandemic's intensity, spread, and persistence [139]. The growth rate of the Indian economy, for instance, slowed to 5% this year from 7% in 2018, the slowest pace in a decade. Rural consumer growth at zero, employment at a 40‐year high, and no progress in the manufacturing sector all contribute to a stagnant economy. Hence, a shutdown would have halted industrial production, leading to an economic collapse [140]. The government of India and the Reserve Bank of India's combined efforts will help in changing the current economic scenario and boosting market growth [139]. The world's stock exchanges have experienced a sharp decline in value. Governments throughout the world have enacted a variety of economic, fiscal, and monetary policies to mitigate the fallout from the economic crisis brought on by COVID‐19 [141].

### 5.3 Health and sociopsychological impacts

The worsened global pandemic situation has left a negative impact on human health. India is severely affected due to strict nationwide disease intervention policies, such as lockdown and burden of restriction; people are scared and mentally traumatized. Daily wage labors, farmers, low‐income families, and migrated citizens are stressed and filled with anxiety due to the unavailability of income sources, unemployment, scarcity of two‐time meals, facilities to stay, and primary healthcare issues [142]. Healthcare professionals were engaged in the service of treating COVID‐19, and staff and patients were experiencing community negligence due to social stigma or fear. To prevent such circumstances, the WHO issued a report addressing mental health considerations to manage the psychosocial well‐being of all citizens [143]. To circumvent such behavioral health issues, ICMR and MoHFW mutually address the people to manage stress and anxiety [144] and awareness regarding the social stigma related to COVID‐19 [145]. Social restrictions are enforced at malls, schools, companies, workplaces, and entertainment areas, limiting the public movement space. COVID‐19 can also spread when people travel long distances to visit family during religious holidays, especially if they take public transportation between cities [146]. Based on variables including the number of COVID‐19 cases and the rate at which COVID‐19 cases doubled, the red, orange, and green zone classification was determined. The economic costs of violence against women range from 1% to 4% of the world's gross domestic product. Nonetheless, rape and sexual assault complaints fall in areas with the strictest lockdowns, which was in line with a reduction in the mobility of women in public areas, on public transportation, and at work, where they may be more vulnerable to rape and sexual assault [147].

### 5.4 Impact on education

The widespread pandemic has led to a temporary shutdown of many schools, academic institutions, universities, and research institutions across the globe [148]. Indian schools, colleges, and universities were closed but many healthcare R&D institutions working on COVID‐19‐related research. The Ministry of Human Resource Development has endorsed digital learning programs (online education) so as to reduce educational loss and streamline the process amid the lockdown. Almost 200 million school employees and 1.6 billion students were unable to attend school during the COVID‐19 health crisis because many schools had to remain closed while educational procedures had to be changed. Around 60 million instructors will be forced to participate in online learning to maintain some semblance of education if academic institutions that still exist are not drastically reorganized. Obviously, the rapid adoption of remote instructional techniques and altered school management procedures posed a challenge to the pedagogic foundation of education, requiring both teachers and students to quickly modify their pedagogies and teaching methodologies [149]. A major drawback of the online mode of attending classes is that economically stable parents can afford the requirements of the students but lower‐income parents are still a question mark for this question. Along with this, online modes of teaching and attending classes have an impact on students who are physically and mentally disabled [150].

### 5.5 Impact on food chain, agriculture, and transport

The disruptions caused by the COVID‐19 pandemic have had a significant impact on the food supply chains of many countries, according to food consumption patterns and other political, economic, and social considerations [151]. Nearly, 820 million people in the world were experiencing chronic hunger, whereas 113 million are dealing with critical severe hunger insecurity. There are drastic consequences of COVID‐19 on food insecurity in around 44 countries and they need external assistance for food supplies [152]. Due to the perishability of the majority of agricultural products in Iran, particularly summer crops, greenhouse goods, flowers, and ornamental plants, as well as the drop in demand brought on by the financial crisis, many of these goods go unused and are lost. In Liberia, 47% of farmers said they were unable to cultivate their fields due to the prevalence of corona illness. Moreover, regulations that were in place made it difficult to harvest goods for the fall. The likely effects of this pandemic on food production, particularly in key importing and exporting nations like the United States, China, and the European Union, may have serious repercussions for global food prices and access. Global food security and nutrition faced enormous challenges as a result of this predicament [153]. The food supply cycle is complex inclusive of producer, consumer, distributor, dairy and fisheries, meat market, grains, and vegetables [154]. Their warehousing, transport, and marketing‐related sectors will be affected immensely. The nations with the highest diffusion of COVID‐19 will receive the worst impact on food supply, agriculture, FMCG products, and essential services like transportation and marketing. The majority of transportation services have not been subject to controlled closures despite being acknowledged as being crucial to food supply networks. To safeguard both their workers and their clients, they have nevertheless implemented a number of social distance protocols.

Thankfully, spatially dispersed labor deployment in current transportation systems often requires little to no face‐to‐face interaction. The variable cost of trucking has decreased as a result of the 15%–20% decline in diesel prices [155].

## 6 COVID‐19: ANALYZING GLOBAL RESPONSES AND STRATEGIES FOR FUTURE PREPAREDNESS

The COVID‐19 pandemc has seen a wide range of responses from different countries, races, and cultures, reflecting the diversity of approaches and perspectives around the world. These varied responses have been influenced by factors such as healthcare infrastructure, government policies, cultural norms, and societal attitudes. According to a new UN report, South Korea has had the most effective response and the United Kingdom had the worst [156]. In a broad and in‐depth portrait of how different nations responded to the same pandemic, the few countries that were relatively successful by the end of 2020 were alike in regard to their responses. These “happy countries”—such as South Korea, Vietnam, New Zealand, and Denmark—had a fast public health response [157]. Let uss explore some illustrations to provide a detailed understanding:

a. Government policies and strategies:
  - *New Zealand's response*: New Zealand implemented early and strict measures, including closing borders, implementing widespread testing and contact tracing, and enforcing strict lockdowns. Their proactive approach resulted in the successful containment of the virus and low case numbers [158, 159, 160].
  - *Sweden's approach*: Sweden took a different approach by opting for less stringent lockdown measures and relying more on voluntary guidelines. Their strategy aimed to achieve herd immunity naturally. However, this approach faced criticism and resulted in a higher infection and mortality rate compared to neighboring countries [161, 162].
b. Healthcare infrastructure:
  - *Germany's testing capabilities*: Germany's robust healthcare system allowed for extensive testing capacities. They were able to conduct widespread testing, including asymptomatic individuals, which aided in early detection and containment efforts [163].
  - *Resource limitations in developing countries*: Some developing nations faced significant challenges due to limited healthcare infrastructure, inadequate testing capabilities, and a shortage of medical supplies. These limitations hampered their ability to respond effectively and control the spread of the virus [164].
c. Cultural norms and behaviors:
  - In many Asian countries, such as Japan and South Korea, wearing masks during outbreaks or when feeling unwell is a common practice rooted in cultural norms. This cultural acceptance and adherence to mask‐wearing contributed to effective containment measures [165, 166, 167].
  - In some countries, cultural values emphasizing personal freedom and individualism resulted in resistance to government‐imposed restrictions. This resistance sometimes led to noncompliance with guidelines, making containment efforts more challenging [166, 168, 169].
d. Public awareness and understanding:
  - *Public health campaigns*: Several countries, such as Australia and Canada, launched extensive public health campaigns to educate the public about COVID‐19, including proper hygiene practices, social distancing, and the importance of vaccination. These campaigns played a crucial role in raising awareness and promoting preventive measures [170, 171].
  - *Misinformation challenges*: The rapid spread of misinformation and conspiracy theories posed significant challenges to public health efforts. Some individuals, influenced by false information, questioned the severity of the virus or the safety of vaccines, hindering effective responses [172, 173].

These illustrations demonstrate the diverse range of responses and the influence of various factors on countries, races, and cultures during the pandemic. It highlights the importance of understanding these differences to learn from successful approaches, address shortcomings, and develop more effective strategies for future uncertainties. By sharing knowledge and collaborating globally, we can strive to enhance preparedness and response capabilities, ultimately mitigating the impact of future crises.

## 7 CONCLUSIONS

After 3 long years of COVID‐19, a lot has changed and many things are yet to be changed. Science has developed and advanced research medicine is heading toward the future of the next generations but there is no standard care given for COVID‐19‐like catastrophes. Thus far, we have only developed preventative strategies for COVID‐19; no cure has been found till date. COVID‐19 cases might have declined but its influence on the society is still stagnant. Many are suffering from mental, physical, social, and financial trauma post‐COVID. Prevention and management and immunization efforts are appreciable but there is a need to improve by investing in science and technology for the healthcare sector. It took around 3 years to curb the pandemic partially with loads of losses. During COVID‐19 India emerged as a pharmacy of the world helping many countries to eradicate the pandemic with tonnes of vaccine doses. Still, many developing countries are struggling with the pandemic. However, it will take longer to overcome COVID‐19‐wide impacts. On the contrary, the pandemic time has given newer trends like digital currencies and digital learning platforms but simultaneously poured some with poor practical abilities. Many are suffering from financial losses and nonreversible health issues. Geopolitical tensions are heightened between global leaders. This experience teaches us that, even though we emerged from a cloud of darkness, there is still hope ahead and we have to move forward with a vision to make our future better for the generations to come.

## AUTHOR CONTRIBUTIONS

**Amol Chhatrapati Bisen**: Conceptualization (lead); data curation (lead); formal analysis (lead); writing—original draft (lead); writing—review and editing (lead). **Sristi Agrawal**: Formal analysis (supporting); methodology (supporting). **Sachin N. Sanap**: Investigation (supporting); methodology (supporting). **Heamanth Ganesan Ravi Kumar**: Methodology (supporting); writing—review and editing (supporting). **Nelam Kumar**: Investigation (supporting); methodology (supporting). **Rajdeep Gupta**: Methodology (supporting). **Rabi Sankar Bhatta**: Project administration (lead); resources (lead); supervision (lead); validation (lead).

## CONFLICT OF INTEREST STATEMENT

The authors declare no conflict of interest.

## ETHICS STATEMENT

Not applicable.

## INFORMED CONSENT

Not applicable.

## Data Availability

Data sharing is not applicable as no new data was generated.
